# Development of an Electrochemical Biosensor for the Detection of Aflatoxin M_1_ in Milk

**DOI:** 10.3390/s101009439

**Published:** 2010-10-20

**Authors:** Nathalie Paniel, Antonio Radoi, Jean-Louis Marty

**Affiliations:** Laboratoire IMAGES EA 4218, Bâtiments S, Université de Perpignan Via Domitia, 52 Avenue Paul Alduy, 66860 Perpignan Cedex, France

**Keywords:** electrochemical immunosensor, aflatoxin M_1_, mycotoxin, milk, horseradish peroxidase (HRP), superparamagnetic nanoparticles

## Abstract

We have developed an electrochemical immunosensor for the detection of ultratrace amounts of aflatoxin M_1_ (AFM_1_) in food products. The sensor was based on a competitive immunoassay using horseradish peroxidase (HRP) as a tag. Magnetic nanoparticles coated with antibody (anti-AFM_1_) were used to separate the bound and unbound fractions. The samples containing AFM_1_ were incubated with a fixed amount of antibody and tracer [AFM_1_ linked to HRP (conjugate)] until the system reached equilibrium. Competition occurs between the antigen (AFM_1_) and the conjugate for the antibody. Then, the mixture was deposited on the surface of screen-printed carbon electrodes, and the mediator [5-methylphenazinium methyl sulphate (MPMS)] was added. The enzymatic response was measured amperometrically. A standard range (0, 0.005, 0.01, 0.025, 0.05, 0.1, 0.25, 0.3, 0.4 and 0.5 ppb) of AFM_1_-contaminated milk from the ELISA kit was used to obtain a standard curve for AFM_1_. To test the detection sensitivity of our sensor, samples of commercial milk were supplemented at 0.01, 0.025, 0.05 or 0.1 ppb with AFM_1_. Our immunosensor has a low detection limit (0.01 ppb), which is under the recommended level of AFM_1_ [0.05 μg L-1 (ppb)], and has good reproducibility.

## 1. Introduction

Aflatoxins are a group of secondary metabolites produced by fungi. Different aflatoxins exist, including aflatoxins B_1_, B_2_, G_1_ and G_2_. Aflatoxin B_1_ is mainly produced by two fungi, *Aspergillus flavus* and *Aspergillus parasiticus* [1,2]. These fungi grow on a great variety of food commodities under a variety of temperature and humidity conditions, and contamination of animal feed materials, including corn, peanuts, cereal crops, either before or after harvest, is a common occurrence [1,3,4]. The optimal growth temperature of mycotoxin-producing moulds ranges between 24 and 35 °C. Crops that grow in warm, humid areas, principally subtropical and tropical countries [5], are contaminated the most often. This contamination results in important losses in terms of human and animal health and agricultural production [6]. Ecological and environmental conditions contribute to the production of mycotoxins in food or feed [7]. Mycotoxins exhibit a wide range of biological effects, and individual mycotoxins can be mutagenic, carcinogenic, embryo-toxic, teratogenic, oestrogenic or immunosuppressive [2].

**Scheme 1.**
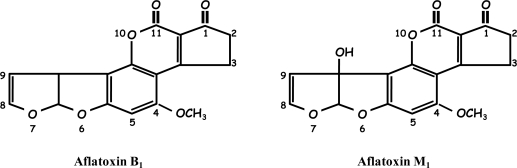
The structures of aflatoxin B_1_ and aflatoxin M_1_. The only difference between the two compounds is the presence of the hydroxyl group at the 9a position of AFM_1_. Both molecules have the 8,9-double bond, which is the putative active site of the molecule [9].

When aflatoxin B_1_ (AFB_1_), the most toxic aflatoxin, is ingested by cows through contaminated feed [2], it is transformed into aflatoxin M_1_ (AFM_1_) through enzymatic hydroxylation of AFB_1_ at the 9a-position (Scheme 1) and has an approximate overall conversion rate equal to 0.3 to 6.2% [1,8,9]. AFM_1_ is secreted in milk by the mammary gland of dairy cows [9,10]. Even though it is less toxic than its parent compound, AFM_1_ has hepatotoxic and carcinogenic effects [4,11]. This toxin, initially classified as a Group 2B agent [12], has now been reclassified as Group 1 by the International Agency for the Research on Cancer (IARC) [13].

AFM_1_ is relatively stable during the pasteurisation, storage and preparation of various dairy products [4,14], and therefore, AFM_1_ contamination poses a significant threat to human health, especially to children, who are the major consumers of milk.

The legal regulations concerning AFM_1_ levels in milk and dairy products vary from country to country. EU regulations allow a maximum level of 0.05 μg L^−1^ (ppb) AFM_1_ in milk [15]. The official methods of sampling and analysis are regulated by the European Commission directive [16]. High-performance liquid chromatography analysis with fluorometric detection (HPLC-FD) coupled with clean-up treatment by immunoaffinity columns (IC) is the reference method used for the determination of aflatoxin concentrations in milk [17]. This procedure, which is long and laborious, requires expensive equipment and well-trained personnel. Other methods for AFM_1_ concentration determination have also been proposed: thin layer chromatography [18], fluorescence detection after immunoaffinity clean-up [19], liquid chromatography coupled to mass spectrometry [20] and immunoenzymatic assays.

To minimise the occurrence of AFM_1_, it is essential to identify the sources of contamination using rapid, selective and sensitive assays. Immunochemical assays, which are rapid, simple, specific, sensitive and even portable, have become the most common quick methods for the routine analysis of mycotoxins in food and feed materials [21,22]. There is a need for more suitable methods, and rapid methods based on the use of biosensors or immunosensors have been proposed in the last decade [23,24]. The aim of our work was to develop a method for aflatoxin M_1_ (AFM_1_) detection and quantification in milk samples using an electrochemical immunosensor. A screen-printed carbon electrode is chosen as the transducer.

## 2. Materials and Methods

### 2.1. Safety notes

Aflatoxins are highly carcinogenic and should be handled with extreme care. Aflatoxin-contaminated labware should be decontaminated with an aqueous solution of sodium hypochlorite (5%). Aflatoxins are subject to light degradation; therefore, analytical work must be protected from daylight, and aflatoxin standard solutions are stored in amber vials. The use of non-acid-washed glassware for aqueous aflatoxin solutions may result in the loss of aflatoxin, and thus special attention should be paid to new glassware. Prior to use, glassware should be soaked in dilute acid (10% sulphuric acid) for several hours and then rinsed extensively with distilled water to remove all traces of acid [25].

### 2.2. Materials and apparatus

The I’Screen AFLA M_1_ milk test kit was from Tecna s.r.l. (Trieste, Italy). Milk samples were obtained from local supermarkets. Aflatoxin M_1_ from *Aspergillus flavus*, 5-methylphenazinium methyl sulphate (MPMS) and hydrogen peroxide (H_2_O_2_) were purchased from Sigma-Aldrich (Germany). Aflatoxin M_1_ linked to horseradish peroxidase (AFM_1_-HRP conjugate) from the I’Screen AFM_1_ milk test kit (Tecna s.r.l, Trieste, Italy) was used. An anti-AFM_1_ antibody (1 mg/mL) was purchased from Soft Flow Biotechnology (Hungary). Superparamagnetic nanoparticles (d = 300 nm), Bio-Adembeads Protein G (uniform-sized superparamagnetic nanoparticles conjugated with protein G), were from Ademtech SA (Pessac, France). Adem-Mag SV (single magnet position adapted for both 1.5/2 mL microfuge tubes or glass vials) were from Ademtech S.A. (Pessac, France). All solutions were stored in glass to limit adsorption. A horizontal shaker (IKA, vibrax, VXR) was also used for the coating step.

Chronoamperometric and cyclic voltammetric measurements were performed with an AUTOLAB PGSTAT12 potentiostat interfaced to a PC, and GPES (General Purpose Electrochemical System) software was used to collect and analyse the data (Utrecht, The Netherlands). DropSens 110 screen-printed carbon electrodes (DropSens, S.L., Spain) were used. We used a three-electrode system, with carbon working and counter electrodes and a silver reference electrode.

### 2.3. Reagents

Phosphate-buffered saline-Tween (PBS-T), 0.05 M, pH 7.4 (Tween-20, 0.05% v/v), and acetate buffer, 0.05 M, pH 5.2, were used.

### 2.4. Preparation of the AFM_1_ standard range and controls

The standard range (0, 0.005, 0.01, 0.025, 0.05, 0.1, and 0.25 ppb) of the AFM_1_ ELISA kit was used. To construct this standard range for AFM_1_, aliquots of the 0 ppb standard milk (blank) from the ELISA kit were spiked with the stock AFM_1_ solution to obtain final concentrations of 0.3, 0.4 or 0.5 ppb. The controls were prepared in PBS-T or in the 0 ppb blank from the ELISA kit. These controls were spiked with the stock AFM_1_ solution to obtain final concentrations of 0.01, 0.025, 0.05 or 0.1 ppb.

### 2.5. Preparation of milk samples

The sample was defatted by centrifugation for 15 min at 6,000 rpm. After centrifugation, the two phases were separated, the fatty cream was discarded, and the skimmed milk was recovered and used to carry out the experimental work. Aliquots of defatted AFM_1_-free milk samples were spiked with the stock solution of AFM_1_ to obtain final concentrations of 0.01, 0.025, 0.05 or 0.1 ppb.

### 2.6. Methods and instrumentation

All affinity reactions were performed off-line by mixing the sample with the tracer (AFM_1_-HRP) and antibody until equilibrium was reached.

### 2.7. Bead preparation

All steps (coating, competition and washing) were carried out with phosphate-buffered saline-Tween (PBS-T), 0.05 M, pH 7.4 (Tween 20 0.05% v/v). Prior to use, the suspended superparamagnetic nanoparticles conjugated with protein G were washed three times with working buffer (26 μL beads + 1374 μL PBS-T) to remove the ProClin 300 which acted as a preservative. The optimised procedure was as follows:

- Coating: the washed beads were collected using the Adem-Mag SV and the antibody solution (2 μg/ml) prepared in working buffer (2.8 μL antibody at 1 mg/mL + 1371 μL PBS-T) was added and allowed to react for 20 minutes. Then, the particles were collected using the Adem-Mag SV, washed three times with working buffer (1,400 μL) and resuspended in 1,400 μL of working buffer.
- A 101-μL aliquot of this dispersion was introduced into a glass vial, and the buffer was removed. Meanwhile, the nanoparticles were collected using the Adem-Mag SV.
- Competition: AFM_1_ (91 μL; from the liquid standard range from the ELISA kit or spiked with milk), AFM_1_-HRP solution (91 μL) prepared in working buffer (1:750 v/v) and acetate buffer (252 μL, 100 mM) were allowed to compete for antibody binding sites for 15–20 minutes. During the coating and competition steps, a horizontal shaker (200 rpm) was employed.

### 2.8. Immunosensor protocol

**Figure 1.**
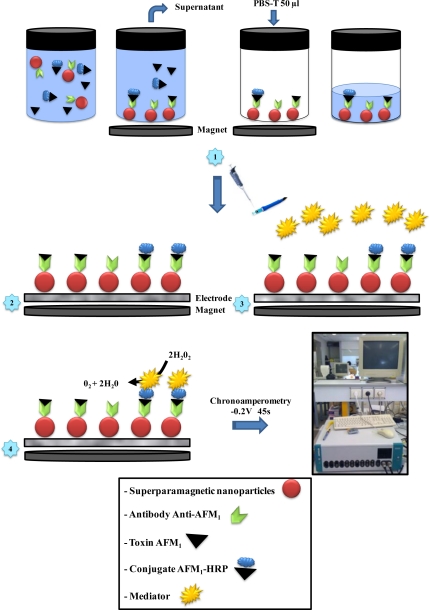
Immunosensor protocol and principle of the electrochemical immunosensor for AFM_1_ detection.

The construction of the immunosensor required the immobilisation of the antibodies on the electrodes via the superparamagnetic nanoparticles. To this end, the screen-printed carbon electrode was placed in a magnet support to collect the superparamagnetic nanoparticles at the electrode surface (Figure 1). Then, after the competition step the particles were collected using the Adem-Mag SV, the supernatant was discarded and 50 μL of PBS-T was added to resuspend the particles (Figure 1, illustration 1), which were then introduced via a Pipetman (Gilson, France) to the surface of the screen-printed carbon electrode. Only the superparamagnetic nanoparticles remain attached to the screen-printed carbon electrode (Figure 1, illustrations 1 and 2).

Next, the electrode surface was washed with 100 μL of the mediator solution (1 mM MPMS; 10 mM H_2_O_2_; 100 mM acetate buffer) to remove all of the toxin or the conjugate that were not attached to the antibody. Before taking the measurements, 100 μL of the mediator solution was introduced to the surface of the electrode (Figure 1, illustrations 3 and 4). The measurements were carried out using a chronoamperometry method at a potential of −0.2 V *vs.* Ag/AgCl for 45 s. All of the experiments were carried out in triplicate in independent assays.

## 3. Results and Discussion

This immunoassay method is based on the use of an AFM_1_-horseradish peroxidase conjugate (AFM_1_-HRP) as a probe. HRP catalyses the oxidation of various hydrogen-donating substrates with hydrogen peroxide to produce oxidised substrate and water. MPMS and H_2_O_2_ were the substrates used to determine HRP activity.

First, the electrochemical behaviour of both MPMS and MPMS_red_ were investigated to optimise the conditions for the determination of HRP activity by amperometry. A cyclic voltammetric investigation of MPMS was carried out using a carbon electrode (DropSens 110). The addition of HRP to a solution containing the two substrates (MPMS and H_2_O_2_) led to the consumption of MPMS and consequently to a decrease in the oxidation current and a increase in the reduction current. A working potential of −0.2 V (−200 mV) *vs.* Ag/AgCl for the measurement of HRP activity was chosen for this study [26]. At this potential, the current was near zero, and no substrate reduction occurred. These conditions were optimal for enzymatic activity determinations when a small amount of product (MPMS_red_) was measured in the presence of a high concentration of substrate.

Before testing the response of the spiked milk samples, a control assay was performed (Table 1) to verify that the AFM_1_ concentration could be detected accurately by the sensor and to determine the amount of interference from the milk matrix during the measurement. For this control, PBS-T and the 0 ppb blank from the ELISA kit were spiked with the AFM_1_ solution to obtain four different sample concentrations: 0.01, 0.025, 0.05 and 0.1 ppb. Electrochemical measurements of the calibration standard solutions prepared in buffer and in milk were made using the immunosensor (Table 1). The response curve for the standard series, the spiked buffer and the spiked milk were identical. Thus, the defatted milk did not affect the measurements.

After this first step, which validated the immunosensor protocol, we performed the second step of our experiment with real milk samples. The milk used for the standard range came from the ELISA kit, as in the first experiment, and experimental milk samples were from commercial sources.

**Figure 2.**
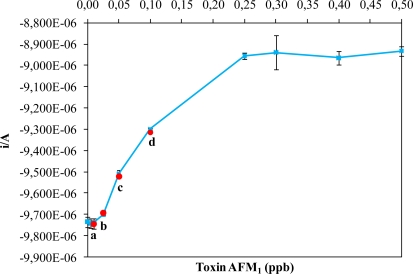
Curve of the AFM_1_ standard range (blue squares) and the spiked AFM_1_ milk samples a, b, c and d (red circles). Vertical bars represent standard errors (not shown when smaller than the symbols).

We constructed a standard curve to determine the relationship between the concentration of AFM_1_ in the sample and the measured intensity. With this standard curve (Figure 2, blue squares), we also calculated the upper and lower limits of detection of the immunosensor. The detection limits of AFM_1_ by the sensor were 0.25 μg L^−1^ (ppb) for the upper limit and 0.01 μg L^−1^ (ppb) for the lower limit (Figure 2 and Table 1).

In the second part of the experiment, commercial milk samples contaminated with a known concentration of AFM_1_ (0.01, 0.025, 0.05 or 0.1 ppb; references a, b, c, d in Table 1 and Figure 2) were tested. The intensity responses for each concentration were measured. The values for the spiked milk samples were the similar to those values measured for the standard range (Table 1 and Figure 2, red circles). The analytical performance of our approach is better for the low concentrations of toxin in comparison with the other. For example, Badea *et al.* [23] realized an flow injection immunoassay system for aflatoxin M_1_ determination and with our approach we have the same limit of detection for the high concentration (0.5 ppb) but we have a higher sensitivity for the lower concentration (0.01 ppb), the same as the system developed by Carlson *et al.* [24].

Our immunosensor allows the detection and the quantification of AFM_1_ over a large range of concentrations. Our immunosensor allows the estimation the real contamination level of spiked milk samples.

## 4. Conclusions

This immunosensor has a working range that is comparable or better than that found for conventional methods. The detection range of 0.01 to 0.1 ppb obtained for milk samples allows the use of this method in dairy industry laboratories. The use of this immunosensor can ensure that the milk purchased by consumers is harmless. Our system allows the measurement of AFM_1_ directly in milk after a single centrifugation step without dilution or pretreatment steps. Another advantage of our method is that the analysis time is reduced and the sample preparation is very simple and fast in comparison with the conventional methods (HPLC and ELISA, for example).

The goal of developing a method using magnetic beads was to optimise this immunosensor by developing a protocol that will allow automation of the sanitary control of foodstuffs. Future work will investigate the development of this immunosensor using flux methods. If the optimisation of a flow-injection system immunoassay for AFM_1_ could be realised, then this assay system would be a good method for the rapid screening of raw milk samples for this toxin. This immunosensor is inexpensive, easy to operate and very suitable to automation.

## Figures and Tables

**Table 1.** Sensor calibration using standard solutions of AFM_1_ and results obtained using control samples and spiked milk.

**AFM_1_ standard range (ppb)**	**Biosensor response (A)**		**Control assays response (A)**				**Spiked milk samples response (A)**		
	**Mean values**	**Standard deviation**	**Spiked PBS-T**	**Standard deviation**	**Spiked 0 ppb ELISA Kit Blank**	**Standard deviation**	**Mean values**	**Standard deviation**	**References**

0	−9.735E-06	2.46E-08							
0.005	−9.743E-06	2.35E-08							
0.01	−9.738E-06	4.93E-09	−9.74E-06	8.60E-08	−9.73E-06	8.35E-08	−9.745E-06	1.617E-08	a
0.025	−9.702E-06	3.06E-09	−9.70E-06	4.00E-08	−9.69E-06	3.91E-08	−9.694E-06	1.528E-09	b
0.05	−9.508E-06	1.48E-08	−9.58E-06	2.98E-08	−9.56E-06	4.68E-08	−9.523E-06	2.442E-08	c
0.1	−9.300E-06	3.61E-09	−9.30E-06	1.89E-08	−9.32E-06	6.64E-08	−9.316E-06	1.106E-08	d
0.25	−8.956E-06	1.53E-08							
0.3	−8.939E-06	7.90E-08							
0.4	−8.965E-06	3.11E-08							
0.5	−8.932E-06	2.28E-08							

*References*:

(a) 0.01 ppb;

(b) 0.025 ppb;

(c) 0.05 ppb;

(d) 0.1 ppb
